# Tetra­kis(μ-4-methyl­benzoato-κ^2^ *O*:*O*′)bis­[(*N*,*N*-diethyl­nicotinamide-κ*N* ^1^)zinc(II)]

**DOI:** 10.1107/S1600536810011517

**Published:** 2010-03-31

**Authors:** Hacali Necefoğlu, Efdal Çimen, Barış Tercan, Hakan Dal, Tuncer Hökelek

**Affiliations:** a Department of Chemistry, Kafkas University, 36100 Kars, Turkey; b Department of Physics, Karabük University, 78050 Karabük, Turkey; c Department of Chemistry, Faculty of Science, Anadolu University, 26470 Yenibağlar, Eskişehir, Turkey; d Department of Physics, Hacettepe University, 06800 Beytepe, Ankara, Turkey

## Abstract

In the centrosymmetric binuclear title complex, [Zn_2_(C_8_H_7_O_2_)_4_(C_10_H_14_N_2_O)_2_], the Zn atoms [Zn⋯Zn′ = 2.9494 (3) Å] are bridged by four 4-methyl­benzoate (PMB) anions. The four nearest O atoms around each Zn^II^ ion form a distorted square-planar arrangement, the octahedral coordin­ation being completed by the pyridine N atom of the *N*,*N*-diethyl­nicotinamide (DENA) ligand. Each Zn^II^ ion is displaced by 0.3530 (1) Å from the plane of the four O atoms. The dihedral angles between carboxyl­ate groups and their adjacent benzene rings are 5.88 (10) and 11.89 (9)°, while the benzene rings are oriented at a dihedral angle of 75.19 (4)°. The pyridine ring is oriented at dihedral angles of 38.28 (4) and 49.17 (4)° with respect to the benzene rings. In the crystal structure, weak inter­molecular C—H⋯O hydrogen bonds link the mol­ecules into a three-dimensional network. π–π contacts between parallel benzene rings [centroid–centroid distance = 3.8388 (8) Å] and between parallel pyridine rings [centroid–centroid distance = 3.4855 (7) Å] may further stabilize the crystal structure.

## Related literature

For niacin, see: Krishnamachari (1974 ▶) and for the nicotinic acid derivative *N*,*N*-diethyl­nicotinamide, see: Bigoli *et al.* (1972 ▶). For related structures, see: Hökelek *et al.* (1995 ▶); Hökelek *et al.* (2009*a* ▶,*b* ▶,*c* ▶); Necefoğlu *et al.* (2010 ▶); Speier & Fulop (1989 ▶); Usubaliev *et al.* (1980 ▶).
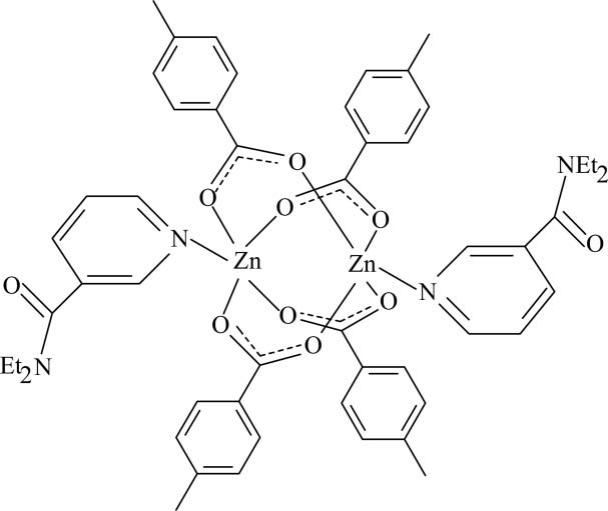


## Experimental

### 

#### Crystal data

[Zn_2_(C_8_H_7_O_2_)_4_(C_10_H_14_N_2_O)_2_]
*M* *_r_* = 1027.82
Triclinic, 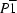

*a* = 9.8603 (2) Å
*b* = 10.5272 (2) Å
*c* = 12.3514 (3) Å
α = 97.346 (3)°
β = 93.525 (3)°
γ = 106.342 (5)°
*V* = 1213.78 (6) Å^3^
*Z* = 1
Mo *K*α radiation
μ = 1.05 mm^−1^
*T* = 100 K
0.45 × 0.35 × 0.34 mm

#### Data collection

Bruker Kappa APEXII CCD area-detector diffractometer
Absorption correction: multi-scan (*SADABS*; Bruker, 2005 ▶) *T*_min_ = 0.649, *T*_max_ = 0.698
22051 measured reflections
6067 independent reflections
5604 reflections with *I* > 2σ(*I*)
*R*_int_ = 0.024

#### Refinement

*R*[*F* ^2^ > 2σ(*F* ^2^)] = 0.024
*wR*(*F* ^2^) = 0.065
*S* = 1.06
6067 reflections
311 parameters
H-atom parameters constrained
Δρ_max_ = 0.50 e Å^−3^
Δρ_min_ = −0.25 e Å^−3^

### 

Data collection: *APEX2* (Bruker, 2007 ▶); cell refinement: *SAINT* (Bruker, 2007 ▶); data reduction: *SAINT*; program(s) used to solve structure: *SHELXS97* (Sheldrick, 2008 ▶); program(s) used to refine structure: *SHELXL97* (Sheldrick, 2008 ▶); molecular graphics: *ORTEP-3 for Windows* (Farrugia, 1997 ▶); software used to prepare material for publication: *WinGX* (Farrugia, 1999 ▶) and *PLATON* (Spek, 2009 ▶).

## Supplementary Material

Crystal structure: contains datablocks I, global. DOI: 10.1107/S1600536810011517/xu2740sup1.cif

Structure factors: contains datablocks I. DOI: 10.1107/S1600536810011517/xu2740Isup2.hkl

Additional supplementary materials:  crystallographic information; 3D view; checkCIF report

## Figures and Tables

**Table 1:** Selected bond lengths (Å)

Zn1—O1^i^	2.0420 (9)
Zn1—O2	2.0264 (9)
Zn1—O3^i^	2.1196 (9)
Zn1—O4	2.0235 (9)
Zn1—N1	2.0340 (10)

Symmetry code: (i) 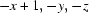.

**Table 2:** Hydrogen-bond geometry (Å, °)

*D*—H⋯*A*	*D*—H	H⋯*A*	*D*⋯*A*	*D*—H⋯*A*
C6—H6⋯O4^ii^	0.93	2.55	3.4601 (18)	166
C16—H16*C*⋯O4^iii^	0.96	2.59	3.542 (2)	174
C19—H19⋯O2^iv^	0.93	2.57	3.4013 (15)	149

Symmetry codes: (ii) 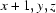; (iii) 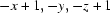; (iv) 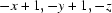.

**Fig. 1.**
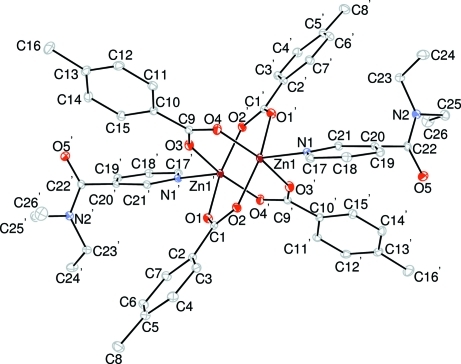
The molecular structure of the title molecule with the atom-numbering scheme. Displacement ellipsoids are drawn at the 30% probability level. Primed atoms are generated by the symmetry operator:(') 1 - x, -y, -z.

## supplementary crystallographic information

### Comment

As a part of our ongoing investigation on transition metal complexes of nicotinamide (NA), one form of niacin (Krishnamachari, 1974), and/or the nicotinic acid derivative *N*,*N*-diethylnicotinamide (DENA), an important respiratory stimulant (Bigoli *et al.*, 1972), the title compound was synthesized and its crystal structure is reported herein.

The title compound is a binuclear compound, consisting of two DENA and four 4-methylbenzoate (PMB) ligands. The crystal structures of similar complexes of Cu^2+^ and Zn^2+^ ions, [Cu(C_6_H_5_COO)_2_(C_5_H_5_N)]_2_ (Usubaliev *et al.*, 1980); [Cu(C_6_H_5_CO_2_)_2_(Py)]_2_ (Speier & Fulop, 1989), [Cu_2_(C_6_H_5_COO)_4_(C_10_H_14_N_2_O)_2_] (Hökelek *et al.*, 1995), [Cu_2_(C_8_H_7_O_2_)_4_(C_6_H_6_N_2_O)_2_] (Necefoğlu *et al.*, 2010), [Zn_2_(C_11_H_14_NO_2_)_4_(C_10_H_14_N_2_O)_2_] (Hökelek *et al.*, 2009a), [Zn_2_(C_8_H_8_NO_2_)_4_(C_10_H_14_N_2_O)_2_].2H_2_O (Hökelek *et al.*, 2009b) and [Zn_2_(C_9_H_10_NO_2_)_4_(C_10_H_14_N_2_O)_2_] (Hökelek *et al.*, 2009c) have also been reported. In these structures, the benzoate ion acts as a bidentate ligand.

The title dimeric complex, [Zn_2_(PMB)_4_(DENA)_2_], has a centre of symmetry and two Zn^II^ ions are surrounded by four PMB groups and two DENA ligands (Fig. 1). The DENA ligands are coordinated to Zn^II^ ions through pyridine N atoms only. The PMB groups act as bridging ligands. The Zn···Zn' distance is 2.9494 (3) Å. The average Zn—O distance is 2.0529 (9) Å (Table 1), and four O atoms of the bridging PMB ligands around each Zn^II^ ion form a distorted square plane. The Zn^II^ ion lies 0.3530 (1) Å above the least-squares plane. The average O—Zn—O bond angle is 88.30 (4)°. A distorted square-pyramidal arrangement around each Zn^II^ ion is completed by the pyridine N atom of DENA ligand at 2.034 (1) Å (Table 1) from the Zn atom. The N1—Zn1···Zn1' angle is 156.05 (4)° and the dihedral angle between plane through atoms Zn1, O1, O2, C1, Zn1', O1', O2', C1' and the plane through Zn1, O3, O4, C9, Zn1', O3', O4' and C9' atoms is 88.08 (9)°. The dihedral angles between the planar carboxylate groups [(O1/O2/C1) and (O3/O4/C9)] and the adjacent benzene rings A (C2—C7) and B (C10—C15) are 5.88 (10) and 11.89 (9) °, respectively, while that between rings A and B is A/B = 75.19 (4)°. Ring C (N1/C17—C21) is oriented with respect to rings A and B at dihedral angles A/C = 38.28 (4) and B/C = 49.17 (4) °.

In the crystal structure, weak intermolecular C—H···O hydrogen bonds (Table 2) link the molecules into a three-dimensional network, in which they may be effective in the stabilization of the structure. The π–π contacts between the benzene rings and between the pyridine rings, Cg2—Cg2^i^ and Cg3—Cg3^ii^, [symmetry codes: (i) 1 - x, -y, 1 - z; (ii) 1 - x, 1 - y, 2 - z, where Cg2 and Cg3 are centroids of the rings B (C10—C15) and C (N1/C17—C21)] may further stabilize the structure, with centroid-centroid distances of 3.8388 (8) and 3.4855 (7) Å, respectively.

### Experimental

The title compound was prepared by the reaction of ZnSO_4_.H_2_O (0.90 g, 5 mmol) in H_2_O (40 ml) and DENA (1.78 g, 10 mmol) in H_2_O (10 ml) with sodium 4-methylbenzoate (1.58 g, 10 mmol) in H_2_O (250 ml). The mixture was filtered and set aside to crystallize at ambient temperature for one week, giving colourless single crystals.

### Refinement

H atoms were positioned geometrically with C—H = 0.93, 0.97 and 0.96 Å, for aromatic, methylene and methyl H atoms, respectively, and constrained to ride on their parent atoms, with U_iso_(H) = xU_eq_(C), where x = 1.5 for methyl H and x = 1.2 for all other H atoms.

### Crystal data

**Table:**

[Zn_2_(C_8_H_7_O_2_)_4_(C_10_H_14_N_2_O)_2_]	*Z* = 1
*M**_r_* = 1027.82	*F*(000) = 536
Triclinic, *P*1	*D*_x_ = 1.406 Mg m^−^^3^
Hall symbol: -P 1	Mo *K*α radiation, λ = 0.71073 Å
*a* = 9.8603 (2) Å	Cell parameters from 9897 reflections
*b* = 10.5272 (2) Å	θ = 2.4–28.4°
*c* = 12.3514 (3) Å	µ = 1.05 mm^−^^1^
α = 97.346 (3)°	*T* = 100 K
β = 93.525 (3)°	Block, colourless
γ = 106.342 (5)°	0.45 × 0.35 × 0.34 mm
*V* = 1213.78 (6) Å^3^	

### Data collection

**Table:**

Bruker Kappa APEXII CCD area-detector diffractometer	6067 independent reflections
Radiation source: fine-focus sealed tube	5604 reflections with *I* > 2σ(*I*)
graphite	*R*_int_ = 0.024
φ and ω scans	θ_max_ = 28.5°, θ_min_ = 1.7°
Absorption correction: multi-scan (*SADABS*; Bruker, 2005)	*h* = −13→13
*T*_min_ = 0.649, *T*_max_ = 0.698	*k* = −14→14
22051 measured reflections	*l* = −16→16

### Refinement

**Table:**

Refinement on *F*^2^	Primary atom site location: structure-invariant direct methods
Least-squares matrix: full	Secondary atom site location: difference Fourier map
*R*[*F*^2^ > 2σ(*F*^2^)] = 0.024	Hydrogen site location: inferred from neighbouring sites
*wR*(*F*^2^) = 0.065	H-atom parameters constrained
*S* = 1.06	*w* = 1/[σ^2^(*F*_o_^2^) + (0.0311*P*)^2^ + 0.5069*P*] where *P* = (*F*_o_^2^ + 2*F*_c_^2^)/3
6067 reflections	(Δ/σ)_max_ < 0.001
311 parameters	Δρ_max_ = 0.50 e Å^−^^3^
0 restraints	Δρ_min_ = −0.25 e Å^−^^3^

### Special details

**Table:**

Geometry. All esds (except the esd in the dihedral angle between two l.s. planes) are estimated using the full covariance matrix. The cell esds are taken into account individually in the estimation of esds in distances, angles and torsion angles; correlations between esds in cell parameters are only used when they are defined by crystal symmetry. An approximate (isotropic) treatment of cell esds is used for estimating esds involving l.s. planes.
Refinement. Refinement of *F*^2^ against ALL reflections. The weighted *R*-factor wR and goodness of fit *S* are based on *F*^2^, conventional *R*-factors *R* are based on *F*, with *F* set to zero for negative *F*^2^. The threshold expression of *F*^2^ > σ(*F*^2^) is used only for calculating *R*-factors(gt) etc. and is not relevant to the choice of reflections for refinement. *R*-factors based on *F*^2^ are statistically about twice as large as those based on *F*, and *R*- factors based on ALL data will be even larger.

### Fractional atomic coordinates and isotropic or equivalent isotropic displacement parameters (Å^2^)

**Table:**

	*x*	*y*	*z*	*U*_iso_*/*U*_eq_	
Zn1	0.450815 (15)	0.115632 (13)	0.040631 (11)	0.01383 (5)	
O1	0.73414 (10)	0.02215 (9)	0.02905 (8)	0.01896 (19)	
O2	0.65938 (9)	0.19668 (9)	0.09519 (8)	0.01795 (18)	
O3	0.49245 (10)	−0.10996 (10)	0.12229 (8)	0.0210 (2)	
O4	0.41059 (10)	0.05856 (9)	0.18860 (8)	0.01957 (19)	
O5	0.30214 (10)	0.44313 (10)	−0.27313 (8)	0.0239 (2)	
N1	0.37721 (11)	0.27588 (10)	0.02830 (9)	0.0147 (2)	
N2	0.08512 (11)	0.30568 (11)	−0.25343 (9)	0.0175 (2)	
C1	0.75162 (13)	0.13428 (12)	0.08691 (10)	0.0154 (2)	
C2	0.89284 (13)	0.19728 (12)	0.15322 (10)	0.0158 (2)	
C3	0.92011 (14)	0.31934 (13)	0.22226 (11)	0.0184 (2)	
H3	0.8532	0.3665	0.2228	0.022*	
C4	1.04631 (14)	0.37058 (13)	0.29001 (12)	0.0212 (3)	
H4	1.0628	0.4518	0.3359	0.025*	
C5	1.14912 (14)	0.30273 (14)	0.29080 (11)	0.0205 (3)	
C6	1.12308 (14)	0.18255 (14)	0.21929 (12)	0.0210 (3)	
H6	1.1913	0.1369	0.2171	0.025*	
C7	0.99677 (14)	0.13047 (13)	0.15144 (11)	0.0193 (3)	
H7	0.9812	0.0503	0.1043	0.023*	
C8	1.28449 (15)	0.35768 (16)	0.36690 (13)	0.0282 (3)	
H8A	1.3353	0.2920	0.3650	0.042*	
H8B	1.2620	0.3787	0.4403	0.042*	
H8C	1.3424	0.4373	0.3439	0.042*	
C9	0.44426 (13)	−0.04751 (13)	0.19693 (10)	0.0165 (2)	
C10	0.42490 (13)	−0.10307 (12)	0.30249 (10)	0.0162 (2)	
C11	0.34787 (14)	−0.05492 (13)	0.38074 (11)	0.0189 (2)	
H11	0.3120	0.0156	0.3692	0.023*	
C12	0.32480 (14)	−0.11202 (15)	0.47572 (11)	0.0224 (3)	
H12	0.2727	−0.0796	0.5270	0.027*	
C13	0.37825 (15)	−0.21695 (14)	0.49557 (11)	0.0235 (3)	
C14	0.45906 (15)	−0.26154 (14)	0.41892 (11)	0.0219 (3)	
H14	0.4984	−0.3294	0.4320	0.026*	
C15	0.48161 (14)	−0.20583 (13)	0.32319 (11)	0.0189 (2)	
H15	0.5350	−0.2373	0.2725	0.023*	
C16	0.34955 (19)	−0.28015 (19)	0.59785 (13)	0.0360 (4)	
H16A	0.3701	−0.3646	0.5892	0.054*	
H16B	0.2515	−0.2941	0.6100	0.054*	
H16C	0.4088	−0.2220	0.6596	0.054*	
C17	0.40194 (13)	0.38549 (13)	0.10388 (10)	0.0160 (2)	
H17	0.4491	0.3876	0.1719	0.019*	
C18	0.35931 (13)	0.49579 (13)	0.08359 (11)	0.0181 (2)	
H18	0.3738	0.5689	0.1384	0.022*	
C19	0.29485 (13)	0.49579 (12)	−0.01931 (11)	0.0173 (2)	
H19	0.2696	0.5704	−0.0357	0.021*	
C20	0.26868 (12)	0.38185 (12)	−0.09764 (10)	0.0152 (2)	
C21	0.30915 (13)	0.27380 (12)	−0.06968 (10)	0.0151 (2)	
H21	0.2883	0.1964	−0.1209	0.018*	
C22	0.21826 (13)	0.37980 (12)	−0.21572 (11)	0.0162 (2)	
C23	−0.01970 (14)	0.23617 (13)	−0.18488 (11)	0.0199 (3)	
H23A	0.0297	0.2137	−0.1231	0.024*	
H23B	−0.0793	0.1532	−0.2275	0.024*	
C24	−0.11255 (15)	0.32037 (15)	−0.14258 (13)	0.0270 (3)	
H24A	−0.1828	0.2694	−0.1020	0.041*	
H24B	−0.1588	0.3458	−0.2034	0.041*	
H24C	−0.0549	0.3993	−0.0955	0.041*	
C25	0.03872 (15)	0.29558 (14)	−0.37030 (11)	0.0218 (3)	
H25A	0.0906	0.3760	−0.3974	0.026*	
H25B	−0.0615	0.2891	−0.3790	0.026*	
C26	0.06320 (19)	0.17447 (16)	−0.43724 (13)	0.0321 (3)	
H26A	0.0298	0.1694	−0.5128	0.048*	
H26B	0.0124	0.0948	−0.4102	0.048*	
H26C	0.1629	0.1824	−0.4311	0.048*	

### Atomic displacement parameters (Å^2^)

**Table:**

	*U*^11^	*U*^22^	*U*^33^	*U*^12^	*U*^13^	*U*^23^
Zn1	0.01471 (8)	0.01387 (7)	0.01481 (8)	0.00699 (5)	0.00123 (5)	0.00289 (5)
O1	0.0180 (4)	0.0163 (4)	0.0221 (5)	0.0056 (3)	−0.0003 (4)	0.0008 (4)
O2	0.0162 (4)	0.0170 (4)	0.0215 (5)	0.0064 (3)	0.0004 (4)	0.0035 (3)
O3	0.0231 (5)	0.0281 (5)	0.0161 (4)	0.0131 (4)	0.0043 (4)	0.0052 (4)
O4	0.0229 (5)	0.0208 (4)	0.0177 (4)	0.0093 (4)	0.0027 (4)	0.0058 (4)
O5	0.0200 (5)	0.0275 (5)	0.0235 (5)	0.0030 (4)	0.0020 (4)	0.0110 (4)
N1	0.0133 (5)	0.0147 (5)	0.0172 (5)	0.0053 (4)	0.0026 (4)	0.0034 (4)
N2	0.0164 (5)	0.0186 (5)	0.0176 (5)	0.0053 (4)	0.0009 (4)	0.0031 (4)
C1	0.0160 (6)	0.0156 (5)	0.0155 (6)	0.0045 (4)	0.0029 (5)	0.0061 (4)
C2	0.0150 (5)	0.0159 (6)	0.0173 (6)	0.0045 (4)	0.0025 (5)	0.0047 (5)
C3	0.0180 (6)	0.0174 (6)	0.0215 (6)	0.0070 (5)	0.0038 (5)	0.0046 (5)
C4	0.0215 (6)	0.0177 (6)	0.0223 (6)	0.0038 (5)	0.0015 (5)	0.0002 (5)
C5	0.0167 (6)	0.0226 (6)	0.0211 (6)	0.0029 (5)	0.0010 (5)	0.0057 (5)
C6	0.0166 (6)	0.0239 (6)	0.0249 (7)	0.0087 (5)	0.0026 (5)	0.0051 (5)
C7	0.0180 (6)	0.0179 (6)	0.0225 (6)	0.0068 (5)	0.0022 (5)	0.0017 (5)
C8	0.0199 (7)	0.0332 (8)	0.0278 (7)	0.0037 (6)	−0.0026 (6)	0.0028 (6)
C9	0.0137 (5)	0.0203 (6)	0.0154 (6)	0.0046 (5)	0.0005 (4)	0.0030 (5)
C10	0.0156 (5)	0.0178 (6)	0.0137 (6)	0.0026 (4)	0.0000 (4)	0.0026 (4)
C11	0.0160 (6)	0.0216 (6)	0.0179 (6)	0.0044 (5)	0.0007 (5)	0.0018 (5)
C12	0.0171 (6)	0.0306 (7)	0.0161 (6)	0.0020 (5)	0.0030 (5)	0.0021 (5)
C13	0.0208 (6)	0.0271 (7)	0.0172 (6)	−0.0027 (5)	−0.0018 (5)	0.0071 (5)
C14	0.0242 (7)	0.0197 (6)	0.0198 (6)	0.0031 (5)	−0.0038 (5)	0.0052 (5)
C15	0.0201 (6)	0.0187 (6)	0.0167 (6)	0.0048 (5)	0.0000 (5)	0.0016 (5)
C16	0.0350 (8)	0.0474 (10)	0.0245 (8)	0.0041 (7)	0.0042 (7)	0.0185 (7)
C17	0.0144 (5)	0.0188 (6)	0.0152 (6)	0.0056 (4)	0.0020 (4)	0.0020 (5)
C18	0.0178 (6)	0.0152 (6)	0.0211 (6)	0.0058 (5)	0.0041 (5)	−0.0012 (5)
C19	0.0165 (6)	0.0145 (5)	0.0233 (6)	0.0073 (4)	0.0048 (5)	0.0041 (5)
C20	0.0116 (5)	0.0162 (5)	0.0185 (6)	0.0045 (4)	0.0023 (4)	0.0043 (5)
C21	0.0142 (5)	0.0137 (5)	0.0173 (6)	0.0045 (4)	0.0011 (4)	0.0014 (4)
C22	0.0171 (6)	0.0135 (5)	0.0201 (6)	0.0078 (4)	0.0009 (5)	0.0031 (5)
C23	0.0152 (6)	0.0200 (6)	0.0231 (6)	0.0025 (5)	0.0012 (5)	0.0051 (5)
C24	0.0217 (7)	0.0266 (7)	0.0328 (8)	0.0061 (5)	0.0088 (6)	0.0040 (6)
C25	0.0214 (6)	0.0239 (6)	0.0184 (6)	0.0056 (5)	−0.0026 (5)	0.0019 (5)
C26	0.0409 (9)	0.0295 (8)	0.0242 (7)	0.0105 (7)	0.0035 (7)	−0.0026 (6)

### Geometric parameters (Å, °)

**Table:**

Zn1—Zn1^i^	2.9494 (3)	C10—C15	1.3934 (18)
Zn1—O1^i^	2.0420 (9)	C11—H11	0.9300
Zn1—O2	2.0264 (9)	C12—C11	1.3876 (19)
Zn1—O3^i^	2.1196 (9)	C12—C13	1.393 (2)
Zn1—O4	2.0235 (9)	C12—H12	0.9300
Zn1—N1	2.0340 (10)	C13—C14	1.393 (2)
O1—Zn1^i^	2.0420 (9)	C13—C16	1.507 (2)
O1—C1	1.2604 (15)	C14—C15	1.3884 (19)
O2—C1	1.2648 (15)	C14—H14	0.9300
O3—Zn1^i^	2.1196 (9)	C15—H15	0.9300
O3—C9	1.2580 (16)	C16—H16A	0.9600
O4—C9	1.2654 (16)	C16—H16B	0.9600
O5—C22	1.2305 (16)	C16—H16C	0.9600
N1—C17	1.3419 (16)	C17—C18	1.3871 (18)
N1—C21	1.3418 (16)	C17—H17	0.9300
N2—C22	1.3433 (16)	C18—H18	0.9300
N2—C23	1.4700 (17)	C19—C18	1.3857 (19)
N2—C25	1.4685 (17)	C19—H19	0.9300
C1—C2	1.5002 (17)	C20—C19	1.3927 (17)
C2—C7	1.3963 (18)	C20—C21	1.3824 (17)
C3—C2	1.3957 (18)	C20—C22	1.5071 (18)
C3—C4	1.3851 (19)	C21—H21	0.9300
C3—H3	0.9300	C23—C24	1.5146 (19)
C4—H4	0.9300	C23—H23A	0.9700
C5—C4	1.3950 (19)	C23—H23B	0.9700
C5—C6	1.3965 (19)	C24—H24A	0.9600
C5—C8	1.5073 (19)	C24—H24B	0.9600
C6—H6	0.9300	C24—H24C	0.9600
C7—C6	1.3870 (19)	C25—C26	1.515 (2)
C7—H7	0.9300	C25—H25A	0.9700
C8—H8A	0.9600	C25—H25B	0.9700
C8—H8B	0.9600	C26—H26A	0.9600
C8—H8C	0.9600	C26—H26B	0.9600
C9—C10	1.4971 (18)	C26—H26C	0.9600
C10—C11	1.3963 (18)		
			
O1^i^—Zn1—O3^i^	84.43 (4)	C13—C12—H12	119.4
O2—Zn1—O1^i^	159.97 (4)	C12—C13—C14	118.42 (13)
O2—Zn1—O3^i^	88.67 (4)	C12—C13—C16	120.61 (14)
O2—Zn1—N1	104.57 (4)	C14—C13—C16	120.97 (14)
O4—Zn1—O1^i^	89.88 (4)	C13—C14—H14	119.6
O4—Zn1—O2	90.22 (4)	C15—C14—C13	120.78 (13)
O4—Zn1—O3^i^	159.93 (4)	C15—C14—H14	119.6
O4—Zn1—N1	107.92 (4)	C10—C15—H15	119.7
N1—Zn1—O1^i^	94.43 (4)	C14—C15—C10	120.52 (13)
N1—Zn1—O3^i^	91.72 (4)	C14—C15—H15	119.7
C1—O1—Zn1^i^	128.83 (8)	C13—C16—H16A	109.5
C1—O2—Zn1	124.35 (8)	C13—C16—H16B	109.5
C9—O3—Zn1^i^	145.07 (9)	C13—C16—H16C	109.5
C9—O4—Zn1	110.41 (8)	H16A—C16—H16B	109.5
C17—N1—Zn1	126.21 (9)	H16A—C16—H16C	109.5
C21—N1—Zn1	114.90 (8)	H16B—C16—H16C	109.5
C21—N1—C17	118.59 (11)	N1—C17—C18	122.00 (12)
C22—N2—C23	124.41 (11)	N1—C17—H17	119.0
C22—N2—C25	118.33 (11)	C18—C17—H17	119.0
C25—N2—C23	117.24 (11)	C17—C18—H18	120.4
O1—C1—O2	125.48 (12)	C19—C18—C17	119.26 (12)
O1—C1—C2	117.03 (11)	C19—C18—H18	120.4
O2—C1—C2	117.47 (11)	C18—C19—C20	118.68 (12)
C3—C2—C1	120.98 (11)	C18—C19—H19	120.7
C3—C2—C7	118.75 (12)	C20—C19—H19	120.7
C7—C2—C1	120.17 (11)	C19—C20—C22	122.14 (11)
C2—C3—H3	119.9	C21—C20—C19	118.57 (12)
C4—C3—C2	120.29 (12)	C21—C20—C22	118.65 (11)
C4—C3—H3	119.9	N1—C21—C20	122.78 (11)
C3—C4—C5	121.29 (12)	N1—C21—H21	118.6
C3—C4—H4	119.4	C20—C21—H21	118.6
C5—C4—H4	119.4	O5—C22—N2	123.51 (12)
C4—C5—C6	118.19 (12)	O5—C22—C20	118.39 (11)
C4—C5—C8	120.84 (13)	N2—C22—C20	118.08 (11)
C6—C5—C8	120.97 (13)	N2—C23—C24	112.25 (11)
C5—C6—H6	119.6	N2—C23—H23A	109.2
C7—C6—C5	120.82 (12)	N2—C23—H23B	109.2
C7—C6—H6	119.6	C24—C23—H23A	109.2
C2—C7—H7	119.7	C24—C23—H23B	109.2
C6—C7—C2	120.62 (12)	H23A—C23—H23B	107.9
C6—C7—H7	119.7	C23—C24—H24A	109.5
C5—C8—H8A	109.5	C23—C24—H24B	109.5
C5—C8—H8B	109.5	C23—C24—H24C	109.5
C5—C8—H8C	109.5	H24A—C24—H24B	109.5
H8A—C8—H8B	109.5	H24A—C24—H24C	109.5
H8A—C8—H8C	109.5	H24B—C24—H24C	109.5
H8B—C8—H8C	109.5	N2—C25—C26	111.44 (12)
O3—C9—O4	124.45 (12)	N2—C25—H25A	109.3
O3—C9—C10	117.63 (11)	N2—C25—H25B	109.3
O4—C9—C10	117.92 (11)	C26—C25—H25A	109.3
C11—C10—C9	120.82 (12)	C26—C25—H25B	109.3
C15—C10—C9	120.25 (12)	H25A—C25—H25B	108.0
C15—C10—C11	118.92 (12)	C25—C26—H26A	109.5
C10—C11—H11	119.9	C25—C26—H26B	109.5
C12—C11—C10	120.13 (13)	C25—C26—H26C	109.5
C12—C11—H11	119.9	H26A—C26—H26B	109.5
C11—C12—C13	121.17 (13)	H26A—C26—H26C	109.5
C11—C12—H12	119.4	H26B—C26—H26C	109.5
			
O1^i^—Zn1—O2—C1	0.43 (18)	O1—C1—C2—C7	−1.65 (18)
O3^i^—Zn1—O2—C1	−69.26 (10)	O2—C1—C2—C3	0.75 (18)
O4—Zn1—O2—C1	90.70 (10)	O2—C1—C2—C7	177.09 (12)
N1—Zn1—O2—C1	−160.69 (10)	C1—C2—C7—C6	−174.62 (12)
O1^i^—Zn1—O4—C9	80.22 (9)	C3—C2—C7—C6	1.8 (2)
O2—Zn1—O4—C9	−79.75 (9)	C4—C3—C2—C1	174.42 (12)
O3^i^—Zn1—O4—C9	6.98 (16)	C4—C3—C2—C7	−2.0 (2)
N1—Zn1—O4—C9	174.84 (8)	C2—C3—C4—C5	0.3 (2)
O1^i^—Zn1—N1—C17	139.95 (10)	C6—C5—C4—C3	1.5 (2)
O1^i^—Zn1—N1—C21	−46.58 (9)	C8—C5—C4—C3	−178.45 (13)
O2—Zn1—N1—C17	−46.43 (11)	C4—C5—C6—C7	−1.6 (2)
O2—Zn1—N1—C21	127.04 (9)	C8—C5—C6—C7	178.28 (13)
O3^i^—Zn1—N1—C17	−135.50 (10)	C2—C7—C6—C5	0.0 (2)
O3^i^—Zn1—N1—C21	37.97 (9)	O3—C9—C10—C11	−167.82 (12)
O4—Zn1—N1—C17	48.64 (11)	O3—C9—C10—C15	10.99 (18)
O4—Zn1—N1—C21	−137.89 (8)	O4—C9—C10—C11	11.89 (18)
Zn1^i^—O1—C1—O2	−15.50 (19)	O4—C9—C10—C15	−169.31 (12)
Zn1^i^—O1—C1—C2	163.13 (8)	C9—C10—C11—C12	176.76 (11)
Zn1—O2—C1—O1	12.25 (18)	C15—C10—C11—C12	−2.06 (19)
Zn1—O2—C1—C2	−166.37 (8)	C9—C10—C15—C14	−177.34 (12)
Zn1^i^—O3—C9—O4	−1.2 (2)	C11—C10—C15—C14	1.49 (19)
Zn1^i^—O3—C9—C10	178.47 (10)	C13—C12—C11—C10	0.5 (2)
Zn1—O4—C9—O3	−1.70 (16)	C11—C12—C13—C14	1.6 (2)
Zn1—O4—C9—C10	178.62 (8)	C11—C12—C13—C16	−178.59 (13)
Zn1—N1—C17—C18	173.27 (9)	C12—C13—C14—C15	−2.2 (2)
C21—N1—C17—C18	0.02 (18)	C16—C13—C14—C15	178.01 (13)
Zn1—N1—C21—C20	−171.23 (9)	C13—C14—C15—C10	0.7 (2)
C17—N1—C21—C20	2.77 (18)	N1—C17—C18—C19	−2.94 (19)
C23—N2—C22—O5	−175.96 (12)	C20—C19—C18—C17	3.09 (19)
C23—N2—C22—C20	5.98 (18)	C21—C20—C19—C18	−0.49 (18)
C25—N2—C22—O5	2.33 (19)	C22—C20—C19—C18	−171.23 (11)
C25—N2—C22—C20	−175.72 (11)	C19—C20—C22—O5	70.73 (16)
C22—N2—C23—C24	92.91 (15)	C19—C20—C22—N2	−111.12 (14)
C25—N2—C23—C24	−85.40 (14)	C21—C20—C22—O5	−100.01 (14)
C22—N2—C25—C26	92.54 (15)	C21—C20—C22—N2	78.14 (15)
C23—N2—C25—C26	−89.04 (15)	C19—C20—C21—N1	−2.52 (18)
O1—C1—C2—C3	−178.00 (12)	C22—C20—C21—N1	168.55 (11)

Symmetry codes: (i) −*x*+1, −*y*, −*z*.

### Hydrogen-bond geometry (Å, °)

**Table:**

*D*—H···*A*	*D*—H	H···*A*	*D*···*A*	*D*—H···*A*
C6—H6···O4^ii^	0.93	2.55	3.4601 (18)	166
C16—H16C···O4^iii^	0.96	2.59	3.542 (2)	174
C19—H19···O2^iv^	0.93	2.57	3.4013 (15)	149

Symmetry codes: (ii) *x*+1, *y*, *z*; (iii) −*x*+1, −*y*, −*z*+1; (iv) −*x*+1, −*y*+1, −*z*.
